# Early Identification of the Non-Transplanted Functional High-Risk Multiple Myeloma: Insights from a Predictive Nomogram

**DOI:** 10.3390/biomedicines13010145

**Published:** 2025-01-09

**Authors:** Yanjuan Li, Lifen Kuang, Beihui Huang, Junru Liu, Meilan Chen, Xiaozhe Li, Jingli Gu, Tongyong Yu, Juan Li

**Affiliations:** Department of Haematology, The First Affiliated Hospital of Sun Yat-sen University, Guangzhou 510080, China; liyj355@mail.sysu.edu.cn (Y.L.); kuanglif@mail.sysu.edu.cn (L.K.); hbhui@mail.sysu.edu.cn (B.H.); liujunru@mail.sysu.edu.cn (J.L.); chenmlan@mail.sysu.edu.cn (M.C.); lixzh36@mail.sysu.edu.cn (X.L.); gujingl@mail.sysu.edu.cn (J.G.); yuty25@mail.sysu.edu.cn (T.Y.)

**Keywords:** multiple myeloma, non-transplant candidates, nomogram, predictive model, M protein decline patterns

## Abstract

**Background**: Patients with multiple myeloma (MM) who have a suboptimal response to induction therapy or early relapse are classified as functional high-risk (FHR) patients and have been shown to have a dismal prognosis. The aim of this study was to establish a predictive nomogram for patients with non-transplanted FHR MM. **Materials and Methods**: The group comprised 215 patients in our center between 1 January 2006 and 1 March 2024. To identify independent risk factors, univariate and multivariate logistic regression analyses were performed, and a nomogram was constructed to predict non-transplant FHR MM. To evaluate the nomogram’s predictive accuracy, we utilized bias-corrected AUC, calibration curves, decision curve analysis (DCA), and clinical impact curves (CIC). **Results**: Multivariate logistic regression demonstrated that younger age at onset, a higher proportion of LDH (more than 220 U/L), pattern A + C of M protein decline patterns, a lower proportion of patients with induction treatment efficacy than VGPR, and those undergoing maintenance therapies were independent risk factors for patients with non-transplanted FHR MM. The AUC scores for the training and internal validation groups were 0.940 (95% CI 0.893–0.986) and 0.978 (95% CI 0.930–1.000). DCA and CIC curves were utilized to further verify the clinical efficacy of the nomogram. **Conclusions**: We developed a nomogram that enables early prediction of non-transplant FHR MM patients. Younger age at onset, LDH ≥ 220 U/L, an A + C pattern of M-protein decline, and induction therapy efficacy not reaching VGPR are more likely to be FHR MM patients. Patients who do not undergo maintenance therapy are prone to early progression or relapse.

## 1. Introduction

Traditional risk stratification in clinical practice of MM patients has relied heavily on the revised International Staging System (R-ISS), which incorporates high-risk genetic aberrations and other biological markers [1]. However, this system has its limitations, as evidenced by the fact that 5–10% of patients categorized as low-risk by R-ISS stage I experience relapse within a year, with an overall survival (OS) of merely three years [2]. The advent of next-generation sequencing (NGS) has revolutionized the genetic profiling of MM, unveiling gene expression profiles and mutational signatures that delineate high-risk newly diagnosed MM patients, such as the UAMS GEP70 and SKY92 [3,4]. Moreover, clinical features such as the presence of large focal lesions, extramedullary plasmacytomas (EMD), circulating malignant plasma cells (CPC), and renal failure have also been recognized as indicators of a high-risk phenotype [5,6,7,8,9]. Despite these genetic and clinical insights, they have yet to be universally adopted into clinical consensus or practice.

The quest for more refined risk stratification has led to the emergence of the second revision of the ISS (R2-ISS) and the concept of double-hit MM, both designed to identify an even more select group of patients at the extreme end of the risk spectrum [10,11]. However, there remains a pressing need for precise stratification of patients who have normal cytogenetic abnormalities.

A pivotal study has shed light on a cohort of FHR patients who exhibit a suboptimal response to induction therapy, with a disconcerting 40% mortality rate within three years or early progression within 12 months since therapy initiation, culminating in a grim OS of merely 20.2 months [2]. The definition of FHR in this study did not entirely exclude high-risk cytogenetic abnormalities, thus calling into question the true functional risk status of these patients. In response, Soekojo CY et al. have endeavored to refine the definition of FHR, excluding patients with t (14;16), t (4;14), *TP53* bi-allelic inactivation, and 1q21 amplification based on ISS stage III [12]. By harnessing genomic sequencing data and employing machine learning techniques, they have crafted a classifier for FHR patients grounded in the Compass dataset. However, the clinical parameters were notably absent from the dataset utilized in this study, and the genetic features were only partially reflective of R-ISS and double-hit criteria. The applicability and validity of this refined FHR definition in real-world settings are yet to be fully substantiated. Moreover, a number of studies have confirmed that MM patients with suboptimal induction therapy response or early relapse have poor outcomes [13,14,15,16,17,18]. However, these studies mainly focused on patients who underwent transplantation, without separately analyzing non-transplanted patients.

In this study, we have undertaken a retrospective analysis of the clinical characteristics of non-transplanted FHR MM patients as defined by early progression within 12 months since therapy initiation within our patient cohort. Furthermore, by juxtaposing the FHR and standard-risk (SR) groups, we aim to uncover additional high-risk patients who may not be clearly defined by high-risk cytogenetic abnormalities. This comparative analysis promises to refine our understanding of risk stratification and, in turn, inform more personalized and effective treatment paradigms for patients with MM.

## 2. Materials and Methods

### 2.1. Patient Selection

A retrospective analysis was performed on patients in our hematology department at the First Affiliated Hospital of Sun Yat-sen University from 1 January 2006 to 1 March 2024. Following the exclusion of 212 individuals, 215 patients who satisfied the diagnostic standards set by the International Myeloma Working Group (IMWG) were ultimately incorporated into the training group. The inclusion criteria were as follows: (1). met the IMWG diagnostic criteria for MM, (2). newly diagnosed MM (NDMM) with complete clinical information, (3). do the interphase FISH testing. The exclusion criteria were: (1). lost follow-up visits after diagnosis or missed records at the time of diagnosis, (2). follow-up period of more than 12 months, (3). received autologous hematopoietic stem cell transplantation (ASCT). These patients were not considered for ASCT due to their age (60 years or older) and the presence of comorbid conditions, including tuberculosis, arrhythmia, a secondary tumor, and so on.

**Figure 1:**
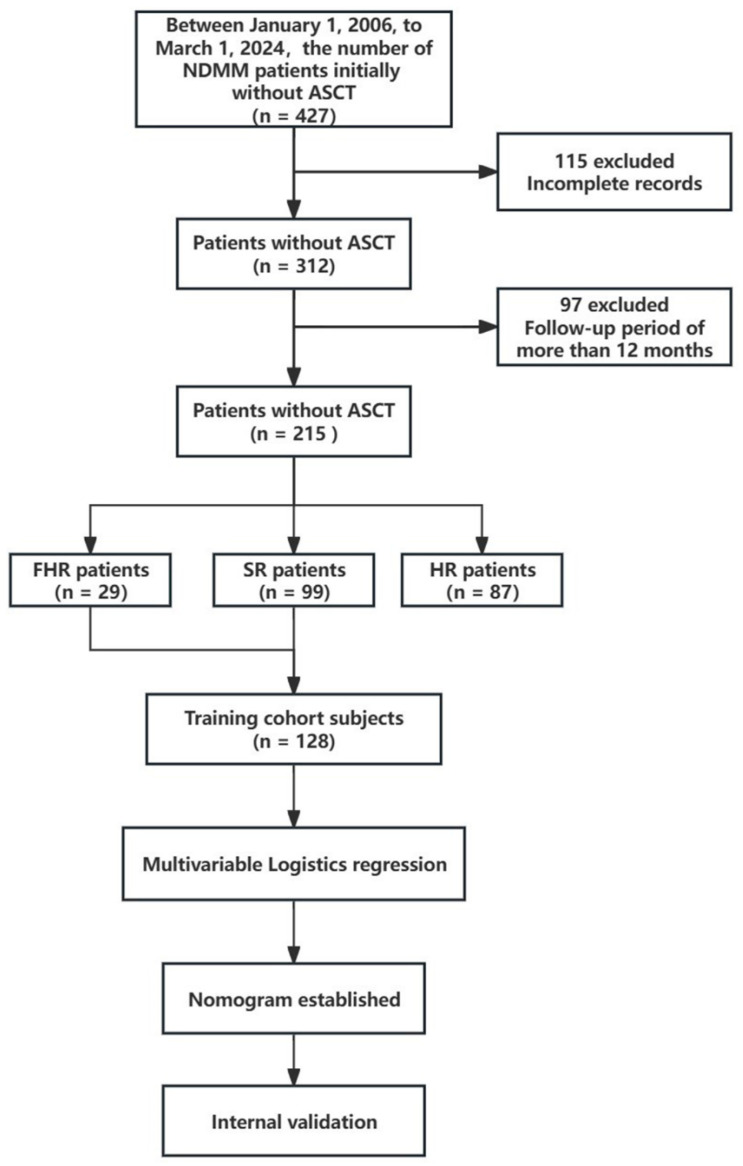
The flowchart of the study.

Non-transplanted FHR MM patients are defined by early progression within 12 months since therapy initiation and do not have high-risk cytogenetics [12]. High-risk (HR) patients are defined as those with high-risk cytogenetics, including del (17p), 1q21 amplification, t (4; 14), t (14; 16), and extramedullary lesions (excluding EMB), and plasma cells can be seen in peripheral blood smear with a percentage of less than 5%. All patients in this study were Chinese. Figure 1 visually illustrates the patient selection process. Criteria and time points for pattern A, B, and C of M protein decline [19]: (1). Pattern A: a serum M-protein decrease ≥90% after 2 cycles of treatment in non-light chain MM cases and a 24-h urine M-protein decrease ≥ 99% after 1 cycle of treatment in light chain MM cases. (2). Pattern B: serum M-protein levels decreasing ≥25% and ˂90% after 2 cycles of treatment in non-light chain MM cases and 24-h urine M-protein levels decreasing ≥50% and not conforming to pattern A after 2 cycles of treatment in light chain MM cases. (3). Pattern C: patients having slow reduction of M-protein after two cycles of treatment, with serum M-protein levels decreasing <25% after 2 cycles of treatment in non-light chain MM cases and 24-h urine M-protein levels decreasing <50% after 2 cycles of treatment in light chain MM cases. Maintenance therapy refers to the continuation of treatment with one or two drugs after 8 cycles of induction therapy. Progression-free survival (PFS) was assessed from the point of the initial diagnosis of MM to either disease progression, death, or the last follow-up. OS was assessed from the point of the initial diagnosis of MM to either death or the last follow-up.

### 2.2. Development and Evaluation of the Nomogram

Univariate logistic regression analysis was conducted to evaluate the relationship among clinical traits. Multivariate logistic regression was used to evaluate independent predictors (entry threshold *p* < 0.050, removal threshold *p* > 0.10), which were subsequently utilized to create a nomogram estimating the likelihood of non-transplanted FHR MM.

Nomogram serves as a valuable instrument by graphically representing estimated probabilities on a 0–100 scale. Points assigned to various covariates represent a patient’s predicted probability. Each variable’s line segment is scaled to show its value range, with the segment length indicating the factor’s impact on the outcome [20].

The total point is the sum of all individual scores after considering all variables. The estimated probability of non-transplanted FHR MM can be obtained by extending a vertical line from the total point axis. The receiver operating characteristic (ROC) curve and the AUC curve were used to assess the ability of the nomogram to distinguish non-transplanted FHR MM. We performed 1000 non-parametric bootstraps and created calibration curves to evaluate the alignment between nomogram prediction and actual observations. DCA curves at different threshold probabilities evaluated the nomogram’s net benefit and clinical utility, offering a novel method for assessing predictive model performance. The CIC visually represents the anticipated number of individuals who will be accurately or inaccurately diagnosed, treated, or classified across various threshold levels. AUC, calibration curves, DCA, and CIC were applied to an internal validation cohort to further assess the nomogram’s stability. AUC, calibration curves, DCA, and CIC were applied to further assess its stability in an internal validation group.

### 2.3. Statistical Analysis

Data analyses were performed by the SPSS Statistics software, version 26 (SPSS Inc, Chicago, IL, USA), and R software version 4.4.1. Data were presented as median with range or as frequency with percentage. The Chi-squared test, or Fisher’s exact test, was utilized for comparing categorical variables, whereas the Mann-Whitney U test was employed for analyzing continuous variables. Multivariate logistic regression analysis was utilized to adjust for significant variables of interest. The Box-Tidwell test was employed to evaluate the linearity assumption in the logit for the continuous variable. The Pearson correlation coefficient was employed to evaluate multicollinearity by examining the variance inflation factor in a multiple regression analysis. The logistic regression model utilized entirely complete data. The multivariable model incorporated only those variables significant to *p* < 0.1 in the univariate models. A *p*-value less than 0.05 in a two-tailed test was deemed to indicate statistical significance.

## 3. Results

### 3.1. Survival of the Three Groups

**Figure 2:**
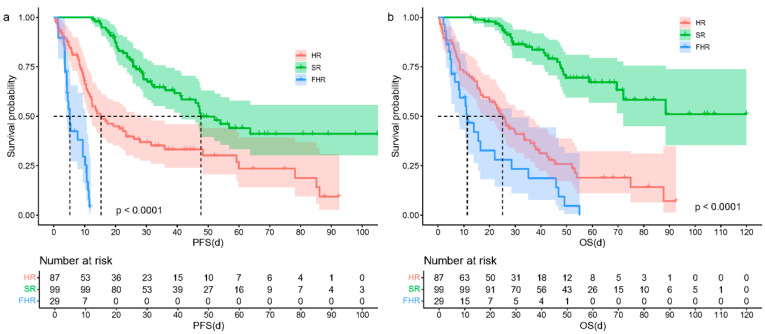
Survival of the three groups. (**a**). PFS of the three groups. (**b**). OS of the three groups. OS of the SR group was not reached. The dashed lines in the figures represent the median survival time for each group.

Based on the definitions of FHR and HR, 215 patients with NDMM were classified into three groups: HR (87 cases, 40.5%), SR (99 cases, 46.0%), and FHR (29 cases, 13.5%). The overall median follow-up time was 45.3 months (95% CI, 37.7–52.9 months). As shown in Figure 2, the OS and PFS of the FHR group were the shortest compared with those of the HR group and the SR group. The FHR group had a median PFS of 5.2 months, compared to 15.3 months for the HR group and 47.7 months for the SR group, with a *p*-value less than 0.001. Regarding median OS, the FHR group had a shorter OS compared to the HR group (11.3 months vs. 24.9 months, *p* = 0.019) and was significantly inferior to that in the SR group, which was not reached (*p* < 0.001).

### 3.2. The Demographic Characteristics of Patients

As Table 1 shows, the age of MM in the FHR group (median age 54 years, range 40–82) was significantly younger compared to the SR group (median age 64 years, range 41–84), with the HR group also showing a younger onset age (median age 63 years, range 44–81). Hemoglobin level did not significantly differ between the FHR and SR groups, but the FHR group showed higher levels compared to the HR group. Platelet count was lower in the FHR group, with a notable difference in PLT counts below 140 when compared to the SR group. However, no significant difference was found between the FHR and HR groups. β2-MG level was notably higher in the HR group compared with the FHR group, but there was no significant difference between the FHR group and the SR group. LDH level was higher in the FHR group compared with the SR group, with a significant difference observed in the percentage of patients with LDH levels greater than or equal to 220, but no significant difference was found between the FHR and HR groups. R-ISS stage showed no significant difference between the FHR and SR groups, with most patients in stages I or II, while the majority in the HR group were in stage III. This suggests R-ISS staging does not effectively differentiate between FHR and SR groups. ECOG score, a measure of performance status, was higher in the FHR and HR groups, indicating a worse functional status in these patients. The ratio of pathological P53 positivity was higher in the FHR group compared to the SR group, although this difference was not statistically significant when compared to the HR group. The folic acid level in the HR group was significantly lower compared to the FHR group. However, no significant difference was observed between the FHR and SR groups. M protein types, serum creatinine level, serum calcium level, ALB level, ISS stage, plasma cell percentage, presence of secondary amyloidosis and EMB (extramedullary bone-related), presence of long term dialysis, vitamin B12 level, and onset blood and urine M protein amount do not significantly vary among the groups.

### 3.3. Treatment Response in Patients

Firstly, as for the decline pattern of M-protein, a statistically significant difference was observed among the three groups (*p* < 0.001). Specifically, Pattern B was identified in 51.7% of patients with FHR, which is significantly lower than the 84.8% observed in the SR group (*p* < 0.001). Pattern C was present in 41.4% of the FHR group, compared to only 3.0% in the SR group and 36.8% in the HR group. Pattern A was the least common, occurring in 6.9% of FHR patients, 12.2% of SR patients, and 13.8% of HR patients. Secondly, within the induction therapy regimen, 89.7% of patients in the FHR group received an induction regimen comprising three drugs. This percentage was higher compared to 73.7% in the SR group and 83.9% in the HR group. However, this difference did not achieve statistical significance (*p* = 0.072). Bortezomib- and ixazomib-based chemotherapy constituted the primary treatment modality, with 75.9% of FHR, 64.6% of SR, and 71.3% of HR patients receiving these agents. Furthermore, regarding induction efficacy, the rates of achieving complete remission (CR) and very good partial remission (VGPR) or better were markedly lower in the FHR group, at 6.9% and 13.8%, respectively, compared to 34.3% and 73.7% in the SR group, respectively, and 13.8% and 35.6% in the HR group (*p* = 0.004 and *p* < 0.001, respectively). Additionally, a significantly smaller proportion of FHR patients received maintenance therapy. All the data are shown in Table 2.

### 3.4. Predictors of FHR

Cases of Pathological P53 positivity ratio were not included in the analysis because of the large number of missing data. As for the univariate analysis, age at diagnosis was significantly lower in the FHR group with a median age of 54 years compared to 64 years in the SR group (*p* < 0.001). Platelet count less than 140 × 10^9^/L was more prevalent in the FHR group, with an odds ratio (OR) of 2.573 (*p* = 0.04). Elevated LDH levels, defined as 220 U/L or higher, were also more common in the FHR group, with an OR of 2.730 (*p* = 0.035). A higher ECOG score of 3 or more was more frequent in the FHR group, with an OR of 2.462 (*p* = 0.036). Induction efficacy up to CR was significantly lower in the FHR group, with an OR of 0.142 (*p* = 0.01). The proportion of patients achieving induction efficacy of at least VGPR was markedly lower in the FHR group, with an OR of 0.057 (*p* < 0.001). Received maintenance therapy was significantly less common in the FHR group, with an OR of 0.07 (*p* < 0.001). M-protein decline pattern B was more frequent in the SR group, with an OR of 0.191 (*p* < 0.001). As for multivariate analysis, age remained a significant factor in differentiating FHR from SR patients, with an adjusted OR of 0.913 (*p* = 0.015). The need for maintenance therapy was also a significant factor, with an adjusted OR of 0.150 (*p* = 0.022). LDH levels of 220 U/L or higher retained their significance with an adjusted OR of 6.318, respectively, although the *p*-value for PLT < 140 × 10^9^/L was 0.079, suggesting a borderline significance. The induction efficacy of at least VGPR and the M-protein decline pattern B continued to show a strong association with the SR group, with adjusted ORs of 0.066 and 0.126 (*p* = 0.005 and *p* = 0.006). Notably, age, LDH levels, ECOG score, induction efficacy, M protein decline pattern, and maintenance therapy were found to be important factors in distinguishing FHR patients from those at standard risk. All the data are shown in Table 3.

### 3.5. Establishment of Predictive Models

**Figure 3:**
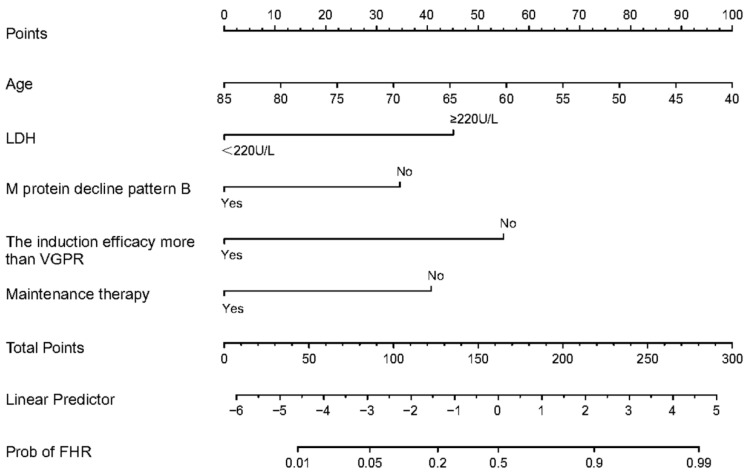
Nomogram for predicting the probability of non-transplant FHR MM. The nomogram can be used by finding each patient’s unique point on each variable axis. To calculate the points that each variable receives, upward-pointing lines and dots are drawn. In order to determine the probability of non-transplant FHR MM, a line is drawn downward from the sum of the points on the Total Points axis.

In the training cohort, patients were randomly divided into the modeling group and the internal validation group in a 7:3 ratio. We identified five predictors of non-transplanted FHR MM, and each factor was scored based on its influence on non-transplanted FHR MM. Figure 3 illustrates a visual nomogram we created to represent the predictive model of non-transplanted FHR MM.

### 3.6. Nomogram Validation

**Figure 4:**
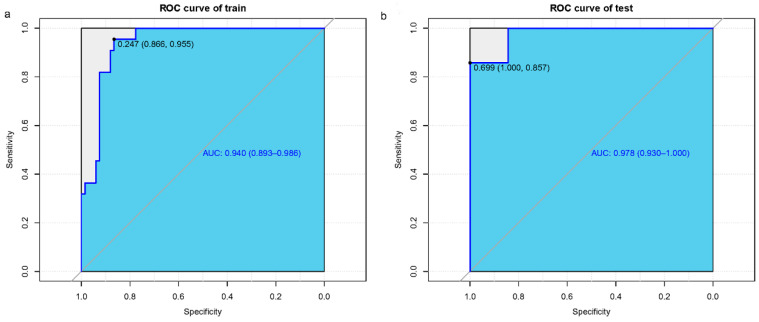
AUC and ROC curves of this nomogram. Training group (**a**) and internal validation group (**b**).

**Figure 5:**
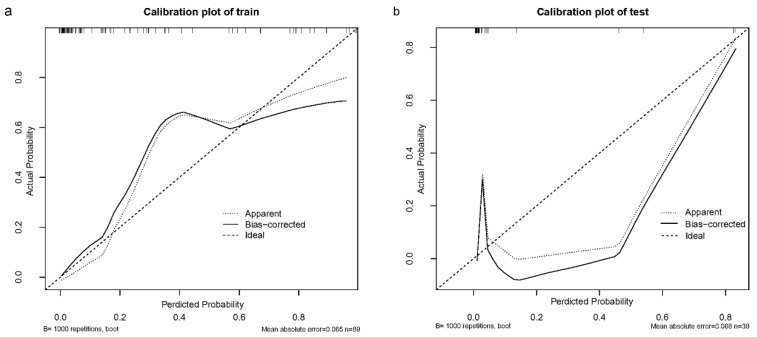
Calibration curves of this nomogram in (**a**) the training group and (**b**) the internal validation group.

**Figure 6:**
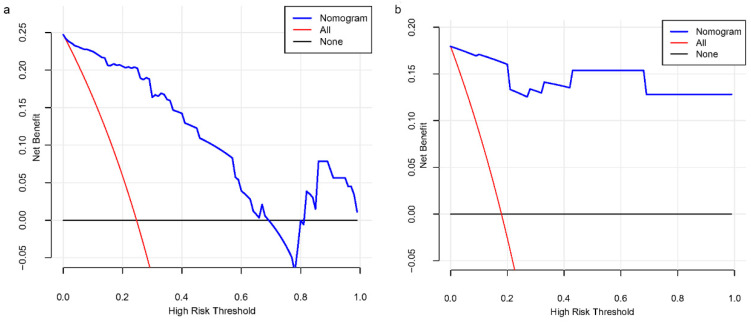
DCA curves of this nomogram in (**a**) the training group and (**b**) the internal validation group.

**Figure 7:**
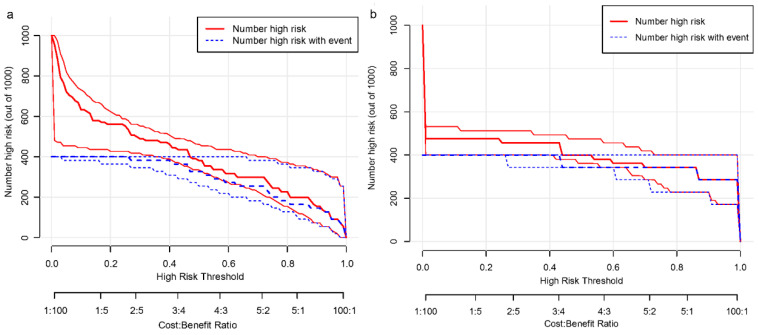
CIC of this nomogram in (**a**) the training group and (**b**) the internal validation group.

For the modeling group, the C-index was 0.940, while the AUC scores were 0.940 (95% CI, 0.893–0.986). For the internal validation group, the C-index was 0.978, and the AUC scores were 0.978 (95% CI, 0.930–1.000). The findings show a consistent degree of precision (Figure 4a,b). Based on the calibration curves, the adjusted bias lines for both the modeling group and internal validation group coincided (Figure 5a,b). Clinical net benefits at different risk thresholds were illustrated by DCA (Figure 6a,b). Threshold ranges of DCA were determined from the modeling group and internal group by evaluating the model’s sensitivity and specificity. Patients with assessed risks within the threshold range were intervened. The net benefit is greater than not intervening or intervening in all patients. Moreover, based on the findings of DCA, we also plotted CIC to evaluate the clinical utility of the nomogram. The CIC of the nomogram showed that the predicted probability coincided well with the actual probability in the modeling group. Similar results were found in the internal validation group (Figure 7a,b).

## 4. Discussion

To the best of our knowledge, this study is the first to establish a predictive nomogram for non-transplanted FHR MM patients in China. FHR MM is a distinct subtype of MM that is typified by its inadequate response to initial induction therapy and a propensity for early relapse. This phenotype presents a significant clinical challenge due to its association with a markedly poor prognosis. The ability to predict the development of FHR MM is critical, as it enables clinicians to make informed decisions regarding the selection and tailoring of treatment strategies. Early identification of patients who are likely to develop FHR MM can lead to the implementation of more intensive or alternative therapeutic approaches, potentially improving patient outcomes and survival rates.

First of all, our study shows the FHR group’s shorter PFS and OS, indicating the aggressive nature of MM in this group, which is similar to other studies [13,14,15,16,17,18]. The younger median age at onset in the FHR group suggests that an earlier manifestation of MM could be indicative of a more aggressive disease progression, necessitating more intensive therapeutic intervention. Our findings additionally revealed a reduced platelet count within the FHR group, consistent with the results reported by Jung SH et al. [21]. The assignment of patients with lower platelet counts to the FHR group could potentially be attributed to the limitation thrombocytopenia imposes on treatment alternatives and the prolonged intervals between chemotherapy sessions it necessitates. Simultaneously, our findings indicated that the levels of LDH and the positive ratios of pathological P53 were elevated in the FHR group. This suggests that patients in the FHR group experienced a higher tumor burden and a more rapid tumor proliferation rate. The ECOG score is a critical indicator of a patient’s functional condition and ability to tolerate therapy. In our study, 55% of patients in the FHR group had ECOG scores greater than 3, suggesting a compromised ability to carry out daily activities and potentially limiting their tolerance of intensive therapy. Other studies have also mentioned that patients with higher ECOG scores have poor prognosis [22,23]. The presence of amyloidosis in MM patients is often linked to a poorer prognosis due to the additional burden of organ involvement and the complexity it adds to the treatment regimen [24]. However, our research data indicate that there is no statistically significant difference in the prevalence of amyloidosis between the FHR group and the SR group, as well as between the FHR group and the HR group. Consequently, it can be inferred that the presence or absence of amyloidosis does not influence the identification of the FHR MM patients. The lower CR and VGPR rates of FHR group also highlight that FHR MM may benefit from more intensive post-induction treatment strategies to delay or prevent disease progression. In addition, we incorporated the M-protein decline pattern from our previous study into a predictive nomogram for the first time [19]. Although patients with pattern A are more likely to achieve remission, they also demonstrate a greater tendency for relapse. Additionally, the evaluation of the M protein reduction pattern occurs after either two or one treatment cycle. A significant number of patients do not quickly reach this evaluative benchmark, resulting in a limited proportion of pattern A patients across the three groups in our study. Our data indicated that, even among non-transplanted MM patients, the A + C pattern of M protein decline was indicative of a poor prognosis. As our data indicates, patients in the FHR group had a lower rate of maintenance therapy, which we hypothesize could be attributed to their early relapse and suboptimal response to initial therapies. This observation aligns with the aggressive nature of FHR MM, where patients often exhibit rapid disease progression and may not achieve sufficient responses to warrant continuation of maintenance therapy.

The multivariate analysis revealed that younger age at onset, higher LDH levels, an A + C pattern of M-protein decline, and lower induction treatment efficacy are independent risk factors for FHR MM. Based on the multivariate analysis results, we developed and validated a predictive nomogram based on readily available clinical data, tailored for non-transplanted FHR MM patients. Our model diverges from traditional risk stratification methods, such as the International Staging System (ISS) and R-ISS [1,25], which incorporate cytogenetic data and are inapplicable to patients with FHR MM. Other prognostic tools for MM are available, which are specifically tailored to either the patient’s functional status, such as the IMWG Frailty Score [26], or to additional disease characteristics, such as the UAMS GEP70 and SKY92 [3,4], which are based on gene expression profiles in MM. While these tools provide valuable prognostic information, outcomes such as OS are influenced by a combination of specific patient, disease, and treatment responses. Our model was able to better predict patients with FHR MM by incorporating a wider range of variables, including age, LDH level, proportion of patients who received maintenance therapy, and treatment response. Multivariate logistic regression analysis ensured that the identified risk factors were independent and provided adjusted odds ratios for each risk factor, providing a more nuanced understanding of their respective effects. By calculating AUC and drawing calibration curves, we have shown that the nomogram has good predictive performance and consistency, showing good predictive value, which is beneficial for predicting non-transplant FHR MM and providing more accurate guidance for intervention and treatment of non-transplant FHR MM patients. In addition, DCA and CIC results indicate that the nomogram has good clinical application value and is conducive to personalized treatment intervention. Comparison with existing literature shows that our study is unique in its emphasis on clinical parameters [5,6,13,14,15,16,17,18]. Although genomic sequencing has advanced the understanding of the MM genetic profile, its clinical application is often limited by accessibility and cost [3,4]. In contrast, our nomogram relies on widely available clinical data, enhancing its practicality and potential for widespread adoption.

However, our study has its limitations. The retrospective design and the predominance of a single ethnic group in the sample may limit the generalizability of our findings, and prospective and multi-ethnic studies may be considered in the future. Additionally, the predictive model’s performance, while robust in our cohorts, must be validated in external, diverse populations to ensure its broad applicability. Future studies should also consider the impact of emerging therapies on the nomogram’s predictive accuracy, as the rapid evolution of MM treatment could influence its long term relevance. Another limitation is the potential for the nomogram to oversimplify the complex interplay of factors influencing MM prognosis. While it offers a comprehensive view within the constraints of current clinical practice, it may not capture the full spectrum of genetic heterogeneity present in MM. Integrating the nomogram with ongoing genomic research could provide a more holistic approach to risk stratification.

## 5. Conclusions

Our study introduces a nomogram that can early predict non-transplanted FHR MM, providing an accurate and user-friendly tool for early risk assessment to guide treatment decisions and improve outcomes with its innovative use of clinical parameters.

## Figures and Tables

**Table 1:** Baseline characteristics of all patients.

Variables	FHR (*n* = 29)	SR (*n* = 99)	*p*-Value	HR (*n* = 87)	*p*-Value
	M (Range)/N (%)	M (Range)/N (%)		**M (Range)/N (%)**	
Sex (%)			0.533		0.912
Males	18 (62.1)	55 (55.6)		55 (63.2)	
Females	11 (37.9)	44 (44.4)		32 (36.8)	
Age (years)	54 (40–82)	64 (41–84)	<0.001	63 (44–81)	<0.001
Type of myeloma (%)			0.337		0.371
IgG	10 (34.5)	49 (49.5)		38 (43.7)	
IgA	5 (17.2)	19 (19.2)		22 (25.3)	
IgD	4 (13.8)	7 (7.1)		4 (4.6)	
IgE	0 (0)	0 (0)		1 (1.1)	
Biclonal	0 (0)	2 (2.0)		1 (1.1)	
Light chain	10 (34.5)	22 (22.2)		21 (24.1)	
Hb (g/L) *	98 (55–160)	92 (49–152)	0.679	86 (35–135)	0.023
PLT < 140 (×10^9^/L) *	11 (37.9)	19 (19.2)	0.036	35 (40.2)	0.872
Serum creatinine (μmol/L)	93 (44–826)	98 (33–1340)	0.900	125 (47–1238)	0.256
Serum calcium (mmol/L)	2.28 (1.9–3.51)	2.3 (1.5–3.86)	0.746	2.3 (1.6–4.26)	0.679
ALB ≤ 35 (g/L) *	16 (55.2)	43 (43.4)	0.265	49 (56.3)	0.914
β2-MG ≥ 3500 (μg/L) *	18 (62.1)	54 (54.5)	0.473	71 (81.6)	0.031
LDH ≥ 220 (U/L) *	10 (34.5)	16 (16.2)	0.031	23 (26.4)	0.406
ISS stage (%)			0.485		0.256
I	4 (13.8)	24 (24.3)		6 (6.9)	
II	11 (37.9)	34 (34.3)		25 (28.7)	
III	14 (48.3)	41 (41.4)		56 (64.4)	
R-ISS stage (%)			0.786		<0.001
I	4 (13.8)	18 (18.2)		2 (2.3)	
II	25 (86.2)	81 (81.8)		36 (41.4)	
III	0 (0)	0 (0)		49 (56.3)	
Plasma cells percentage (%)	22.5 (3–79.5)	20 (1–79.5)	0.368	34.5 (4–94.5)	0.063
ECOG score more than 3 (%) *	16 (55.2)	33 (33.3)	0.033	35 (40.2)	0.663
Presence of secondary amyloidosis (%)	4 (13.8)	15 (15.2)	0.856	12 (13.8)	0.845
Presence of EMB (%) *	12 (41.4)	28 (28.3)	0.223	34 (45.9)	0.641
Long term dialysis (%)	2 (6.9)	5 (5.1)	0.701	6 (6.9)	0.999
Pathological P53 positivity ratio (%)	12/16 (66.7)	6/28 (33.3)	0.002	14/19 (73.7)	0.641
Vitamin B12 (ng/L)	418 (229–1320)	362 (145–1525)	0.451	294 (190–1500)	0.136
Folic acid (μg/L)	16.09 (7.49–25.40)	14.12 (7.24–23.32)	0.480	8.84 (4.77–25.4)	0.011
Onset blood M protein amount (g/L)	32.5 (15.3–109)	37.3 (2.7–126)	0.501	45.5 (1.64–119)	0.695
Onset urine M protein amount (mg/L)	1400.1 (70.35–7224)	1625.4 (7.78–22,035)	0.957	1489.6 (8.9–23,706)	0.918

* Hb = Hemoglobin, PLT = Platelet, ALB = Albumin, β2-MG = Serum β2-microglobulin, LDH = Lactate dehydrogenase, ECOG = The Eastern Cooperative Oncology Group, EMB = Extramedullary bone-related.

**Table 2:** Treatment response in patients.

Variables	FHR (*n* = 29)	SR (*n* = 99)	*p*-Value	HR (*n* = 87)	*p*-Value
	M (Range)/N (%)	M (Range)/N (%)		M (Range)/N (%)	
M-protein decline patterns			<0.001		0.606
A	2 (6.9)	12 (12.2)		12 (13.8)	
B	15 (51.7)	84 (84.8)		43 (49.4)	
C	12 (41.4)	3 (3.0)		32 (36.8)	
B vs. A + C	15 (51.7)	84 (84.8)	<0.001	43 (49.4)	0.83
Induction therapy regimen 1			0.072		0.515
Two drugs	3 (10.3)	26 (26.3)		14 (16.1)	
Three drugs	26 (89.7)	73 (73.7)		73 (83.9)	
Induction therapy regimen 2			0.341		0.566
Based on bortezomib or ixazomib ^a^	22 (75.9)	64 (64.6)		62 (71.3)	
Based on lenalidomide ^b^	4 (13.8)	16 (16.2)		8 (9.2)	
Based on daratumumab and carfilzomib ^c^	2 (6.9)	4 (4.0)		7 (8.0)	
Conventional chemotherapy ^d^	1 (3.4)	15 (15.2)		10 (11.5)	
Induction therapy efficacy					
Reach to CR ^e^	2 (6.9)	34 (34.3)	0.004	12 (13.8)	0.323
More than VGPR ^f^	4 (13.8)	73 (73.7)	<0.001	31 (35.6)	0.026
Received maintenance therapy ^g^	5 (17.2)	74 (74.7)	<0.001	43 (49.4)	0.002

^a^—including VD (Bortezomib, Dexamethasone), PAD (Bortezomib, Liposomal doxorubicin, and Dexamethasone), ID (Ixazomib, Dexamethasone); ^b^—including RD (Lenalidomide, Dexamethasone), RAD (Lenalidomide, Liposomal doxorubicin, and Dexamethasone); ^c^—including DRD (Daratumumab, Lenalidomide, and Dexamethasone), DVD (Daratumumab, Bortezomib, and Dexamethasone), KD (Carfilzomib, Dexamethasone); ^d^—including CTD (Cyclophosphamide, Thalidomide, and Dexamethasone), TD (Thalidomide, Dexamethasone); ^e^—CR = Complete response; ^f^—VGPR = Very good partial response; ^g^—maintenance therapy including VD (Bortezomib, Dexamethasone), Lenalidomide, or Thalidomide.

**Table 3:** General characteristics of the patients and multivariate logistic regression analyses for screening predictors.

Variable	FHR (*n* = 29)	SR (*n* = 99)	Univariate Analysis		Multivariate Analysis	
	M (Range)/N (%)	M (Range)/N (%)	OR	*p*-Value	OR	*p*-Value
Age (years)	54 (40–82)	64 (41–84)	0.919	<0.001	0.913	0.015
PLT < 140 (×10^9^/L)	11 (37.9)	19 (19.2)	2.573	0.04	3.583	0.079
LDH ≥ 220 (U/L)	10 (34.5)	16 (16.2)	2.730	0.035	6.318	0.034
ECOG score more than 3 (%)	16 (55.2)	33 (33.3)	2.462	0.036	3.620	0.067
Induction efficacy up to CR (%)	2 (6.9)	34 (34.3)	0.142	0.01	2.007	0.581
The induction efficacy is more than VGPR (%):	4 (13.8)	73 (73.7)	0.057	<0.001	0.066	0.005
Received maintenance therapy (%)	5 (17.2)	74 (74.7)	0.07	<0.001	0.150	0.022
M protein decline pattern B (%)	15 (51.7)	84 (84.8)	0.191	<0.001	0.126	0.006

## Data Availability

The datasets used and/or analyzed during the current study are available from the corresponding author on reasonable request.
